# Recoverable plasticity in penta-twinned metallic nanowires governed by dislocation nucleation and retraction

**DOI:** 10.1038/ncomms6983

**Published:** 2015-01-13

**Authors:** Qingquan Qin, Sheng Yin, Guangming Cheng, Xiaoyan Li, Tzu-Hsuan Chang, Gunther Richter, Yong Zhu, Huajian Gao

**Affiliations:** 1 Department of Mechanical and Aerospace Engineering, North Carolina State University, Raleigh, North Carolina 27695, USA; 2 School of Engineering, Brown University, Providence, Rhode Island 02912, USA; 3 Centre of Advanced Mechanics and Materials, Applied Mechanics Laboratory, Department of Engineering Mechanics, Tsinghua University, Beijing 100084, China; 4 Max Planck Institute for Intelligent Systems, Heisenbergstrasse 3, D-70589 Stuttgart, Germany

## Abstract

There has been relatively little study on time-dependent mechanical properties of nanowires, in spite of their importance for the design, fabrication and operation of nanoscale devices. Here we report a dislocation-mediated, time-dependent and fully reversible plastic behaviour in penta-twinned silver nanowires. *In situ* tensile experiments inside scanning and transmission electron microscopes show that penta-twinned silver nanowires undergo stress relaxation on loading and complete plastic strain recovery on unloading, while the same experiments on single-crystalline silver nanowires do not exhibit such a behaviour. Molecular dynamics simulations reveal that the observed behaviour in penta-twinned nanowires originates from the surface nucleation, propagation and retraction of partial dislocations. More specifically, vacancies reduce dislocation nucleation barrier, facilitating stress relaxation, while the twin boundaries and their intrinsic stress field promote retraction of partial dislocations, resulting in full strain recovery.

## Abstract (web summary)

1D nanostructures are widely regarded as important building blocks for a broad range of applications. Here, the authors study dislocation-mediated plastic deformation in penta-twinned silver nanowires, finding that *in situ* deformation at small to moderate strains can be entirely reversible.

## Graphical abstract

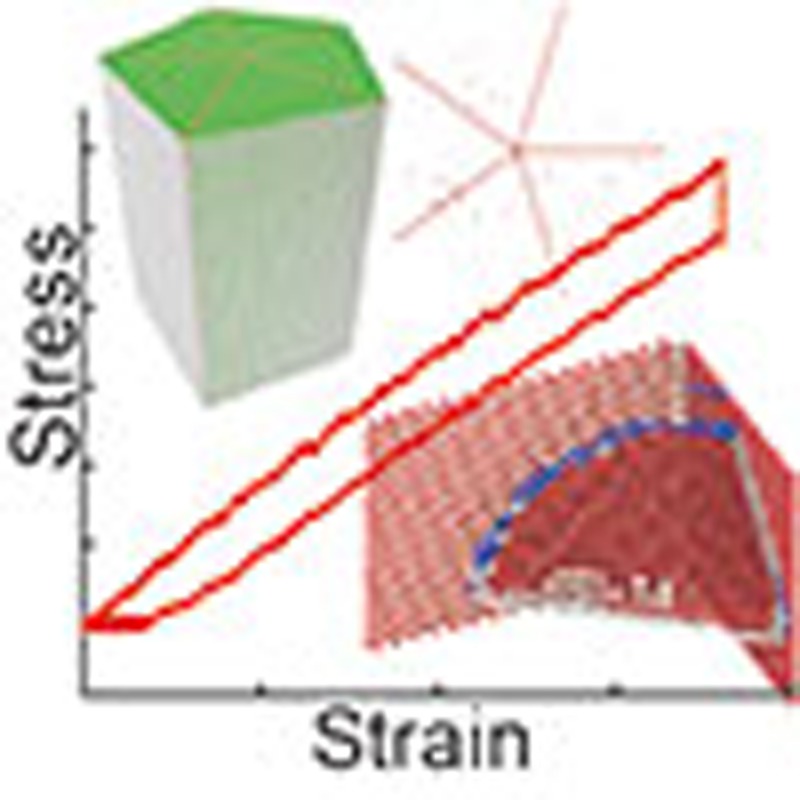

One-dimensional (1D) nanostructures are widely regarded as among the most important building blocks for a broad range of applications including nanoelectronics, optoelectronics, energy harvesting and storage, ultrasensitive sensing and nanoelectromechanical devices^1^,^2^. 1D nanostructures commonly exhibit ultrahigh mechanical strength, which make them also ideal candidates for studying fundamental deformation mechanisms at the nanoscale^3^,^4^,^5^. In the case of metallic nanowires (NWs), dislocation nucleation from free surfaces has been identified as a dominant deformation mechanism, in contrast to the forest dislocation dynamics in bulk materials^6^,^7^,^8^,^9^,^10^,^11^,^12^. Recently, NWs with internal microstructures have received much interest. For instance, metallic NWs with different types of twin boundaries (TBs) have been studied, including parallel, inclined or perpendicular TBs with respect to the NW length direction^13^,^14^,^15^,^16^,^17^,^18^. However, there has been relatively little study on time-dependent responses of NWs under sustained or cyclic loadings, in spite of the obvious importance of this subject to the function and reliability of NW-based devices.

A number of recent experimental and computational studies have revealed substantial time-dependent and partially reversible deformation behaviours in small-scale materials with characteristic length scale below 100 nm^19^, especially nanocrystalline metal thin films^20^,^21^,^22^,^23^. These behaviours have been attributed to the coupling and competition of reversible dislocation activities and grain boundary (GB)-mediated processes at different temperature and strain rates^24^,^25^,^26^,^27^,^28^,^29^,^30^. At relatively high temperatures and low strain rates, GB diffusion/sliding can dominate the time-dependent behaviours, while dislocation nucleation and motion become more prevalent at lower temperatures and higher strain rates. More recently, atomistic simulations predicted a reversible transition between two crystal orientations during loading, leading to shape memory and pseudoelastic behaviours for several face-centred cubic single-crystalline metal NWs^7^,^31^,^32^,^33^. This phenomenon was attributed to the formation of defect-free twins facilitated by relatively low stacking fault energy, nanometer-size scale and surface stress.

Here we report an unusual time-dependent deformation behaviour in penta-twinned Ag NWs, with stress relaxation on loading and complete strain recovery on unloading. Penta-twinned Ag NWs contain five TBs running in parallel to the NW length, exhibiting interesting mechanical properties such as strain hardening^14^,^18^. The critical role of the penta-twinned nanostructure is established by showing that the same phenomenon does not exist in single-crystalline Ag NWs. Large-scale atomistic simulations are then performed to explore the mechanisms underlying the observed behaviours in detail.

## Results

### Structural characterization of Ag NWs

**Figure 1:**
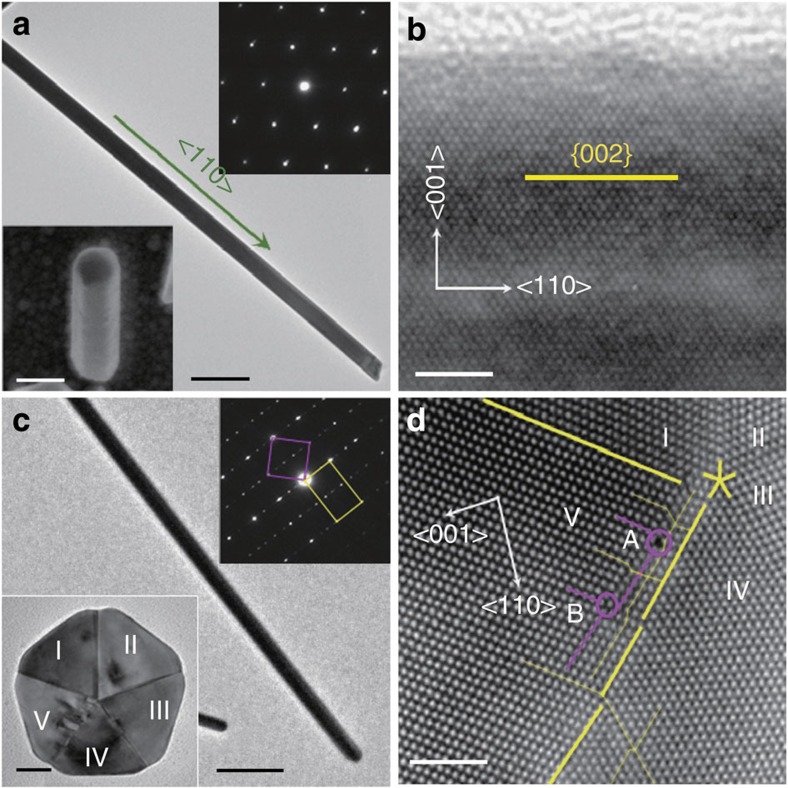
Structural characterization of single-crystal and penta-twinned Ag NWs. (**a**,**b**) Low-magnification and high-resolution TEM images of single-crystal Ag NW with growth direction of <110>. Scale bar, 200 and 2 nm, respectively. Right and left insets (scale bar, 100 nm) in **a** show the selected area electron diffraction (SAED) pattern taken from <110> zone axis and the hexagonal cross-sectional shape from SEM observation, respectively. (**c**) TEM image of Ag NWs showing fivefold twinned structure. Scale bar, 200 nm. Right and left insets (scale bar, 20 nm) in **c** display the corresponding SAED pattern and the pentagonal cross-sectional shape, respectively. Stacking faults along the boundary between grains IV and V can be clearly seen in the left inset of **c**. (**d**) High-angle annular dark-field scanning TEM image of the cross-sectional sample showing the presence of vacancy defects near the boundary between grains IV and V. The yellow star in **d** indicates the centre of the cross-sectional sample. Scale bar, 2 nm.

Microstructure characterization of single-crystalline and penta-twinned Ag NWs is shown in Fig. 1. Both types of NWs are straight and uniform in diameter, with growth direction of <110>, as shown by transmission electron microscopy (TEM) images and selected area electron diffraction patterns in Fig. 1a,c. The single-crystalline Ag NWs exhibit a hexagonal cross-sectional morphology (inset of Fig. 1a). Figure 1b shows a high-resolution TEM image of a single-crystalline Ag NW, indicating a perfect atomic structure along the longitudinal direction and a uniform atomic arrangement at {002} surface facets. The penta-twinned Ag NWs contain a fivefold twinned nanostructure with five TBs running along {111} planes in parallel to the longitudinal axis of the NWs and five surface facets along {100} planes with a pentagonal cross-sectional morphology (inset of Fig. 1c)^18^,^34^.

The characteristics of the fivefold twins are illustrated from the cross-sectional view of the left inset of Fig. 1c,d. The five twin variants (TB-separated nanograins) are numbered from I to V. Stacking faults along the TB between twin variants IV and V can be clearly seen (see Supplementary Fig. 1). To further investigate the defect structures around the TBs, atomic-resolution high-angle annular dark-field scanning TEM imaging is shown in Fig. 1d. In addition to the stacking faults, vacancy defects were identified near the TB, such as those marked by solid circles between twin variants IV and V. The average density of the visible vacancy defects was estimated to be ~2.23 × 10^25^ m^−3^ (0.0375 in percentage), which played an important role in our atomistic simulations. The formation of the vacancy defects is likely caused by interaction of partial dislocations during growth of the penta-twinned NWs. For instance, the vacancy defect marked by A can be formed by the interaction of two partials, 
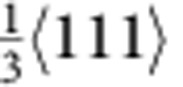
 and 
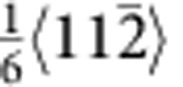
, based on the following reaction, 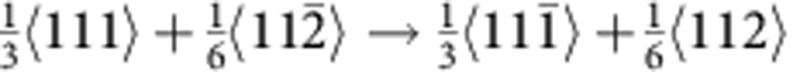. The 
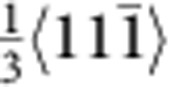
 partial is then locked, but the new 
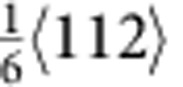
 partial continues to move and interacts with another 
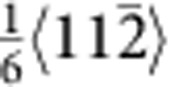
 partial. As a result, a cascade of vacancy defects (for example, the one marked by B in Fig. 1d) can be formed.

### *In situ* tensile testing of Ag NWs

**Figure 2:**
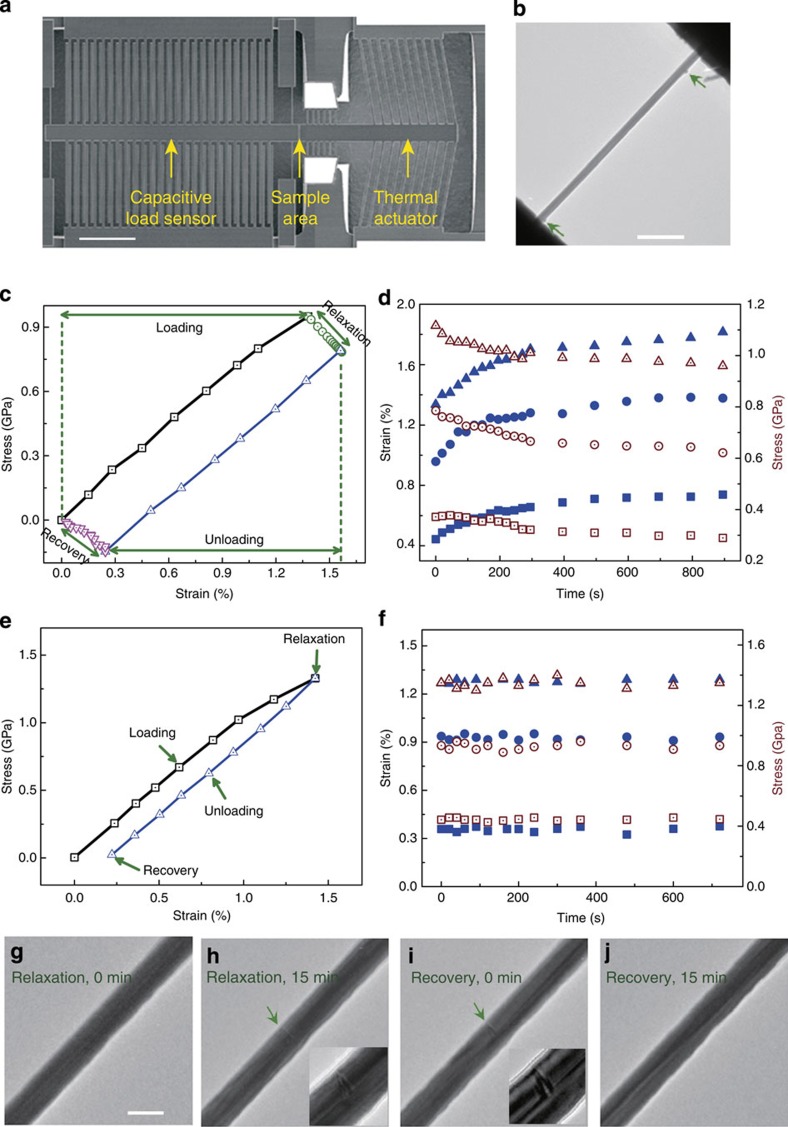
*In situ* measurements of stress and strain evolutions in Ag NWs. (**a**) The MEMS stage used for *in situ* SEM and TEM tensile testing of Ag NWs. Scale bar, 200 nm. (**b**) Low-magnification TEM image of a NW bridged between the actuator and the load sensor, with two marks (arrowed) for displacement measurement. Scale bar, 500 nm. (**c**,**e**) Stress–strain curves for a penta-twinned Ag NW (120 nm in diameter) and a single-crystalline Ag NW (99 nm in diameter). Note that in both cases the relaxation and recovery steps took 15 min each. (**d**,**f**) Relaxation curves for a penta-twinned Ag NW (104 nm in diameter) and a single-crystalline Ag NW (71 nm in diameter). Note: solid and open symbols correspond to the strain–time and stress–time relationships, respectively. Square, circle and triangle symbols correspond to first, second and third stress levels, respectively. (**g**–**j**) A series of TEM images extracted from Supplementary Movies 2 and 3 showing dislocation nucleation and annihilation in a penta-twinned Ag NW (85 nm in diameter). The arrows in **f**,**g** point to the dislocation networks with high-magnification images shown in the insets. Scale bar, 150 nm.

We performed *in situ* tensile experiments inside scanning and transmission electron microscope (SEM/TEM) using a microelectromechanical system (MEMS)-based testing system that allows accurate measurement of both load and displacement simultaneously^35^,^36^,^37^,^38^, as shown in Fig. 2a. Load is applied using a thermal actuator on one side of the testing stage and measured using a differential capacitive sensor on the other side. Details on the load sensor calibration have been reported previously^38^. Displacement (and strain) is measured by digital image correlation of SEM images of two local markers on the specimen. This MEMS-based system has a strain resolution of 0.01% (gage length 2 μm) and a stress resolution of 1.4 MPa (for example, for NW diameter of 104 nm). First, stress relaxation experiments were conducted by loading the thermal actuator to a given displacement and while the actuator displacement was held constant, the specimen relaxed as a function of time. Since the experiment was not under true displacement control, the load on the specimen decreased and the specimen elongation increased at the same time. Subsequently, complete strain recovery was observed after unloading when turning off the thermal actuator. Note that the temperature rise at the specimen end due to the thermal actuator was less than 5 °C for all the experiments reported here^37^.

Figure 2c shows the stress–strain response during a typical tensile test of a penta-twinned Ag NW in four steps: loading, relaxation, unloading and recovery (see also Supplementary Movie 1). During the loading step, the NW exhibited nearly linear response (slightly nonlinear when the applied stress is high). During the relaxation step, the stress decreased with time, while the strain increased. Figure 2d shows the strain and stress as functions of time at three stress levels. When the initial stress is ~0.37 GPa, the NW strain increased monotonically with time from 0.44 to 0.74%, while the stress decreased monotonically from 371 to 290 MPa. On unloading, the strain in the NW completely recovers in about the same amount of time as the relaxation. The same procedure was repeated for the relaxation and recovery steps at two additional stress levels (with initial stresses of 0.79 and 1.12 GPa), corresponding to the circle and triangle symbols in Fig. 2d, respectively.

*In situ* TEM tensile testing indicated that the stress relaxation and strain recovery in a penta-twinned Ag NW are accompanied by nucleation and annihilation of dislocations, respectively, as shown in Fig. 2g–j. Before the relaxation step (Fig. 2g), there were no dislocations in the NW as the strain increased to 1.6%. At the relaxation step, a dislocation network appeared in <5 min (see Supplementary Movie 2). The dislocations were terminated at the TBs (Fig. 2h). During the unloading step, the applied load gradually decreased to zero but the dislocations remained (Fig. 2i). In the recovery step, the dislocation network gradually retracted and suddenly disappeared after ~8 min (Fig. 2j). After 15 min, the NW was totally recovered (see Supplementary Movie 3). Overall, the same relaxation and recovery behaviours were observed in all five penta-twinned Ag NWs with different diameters (48, 85, 104, 120 and 121 nm) tested in either SEM or TEM (see Supplementary Fig. 2).

The same experiments were conducted for five single-crystalline Ag NWs with diameters of 71, 99, 111, 130 and 152 nm at similar stress levels for the penta-twinned Ag NWs, but neither stress relaxation (Fig. 2e,f) nor recovery of plastic strain (Fig. 2f) was observed in any of them (see Supplementary Movie 4).

### MD simulations of stress relaxation and strain recovery

**Figure 3:**
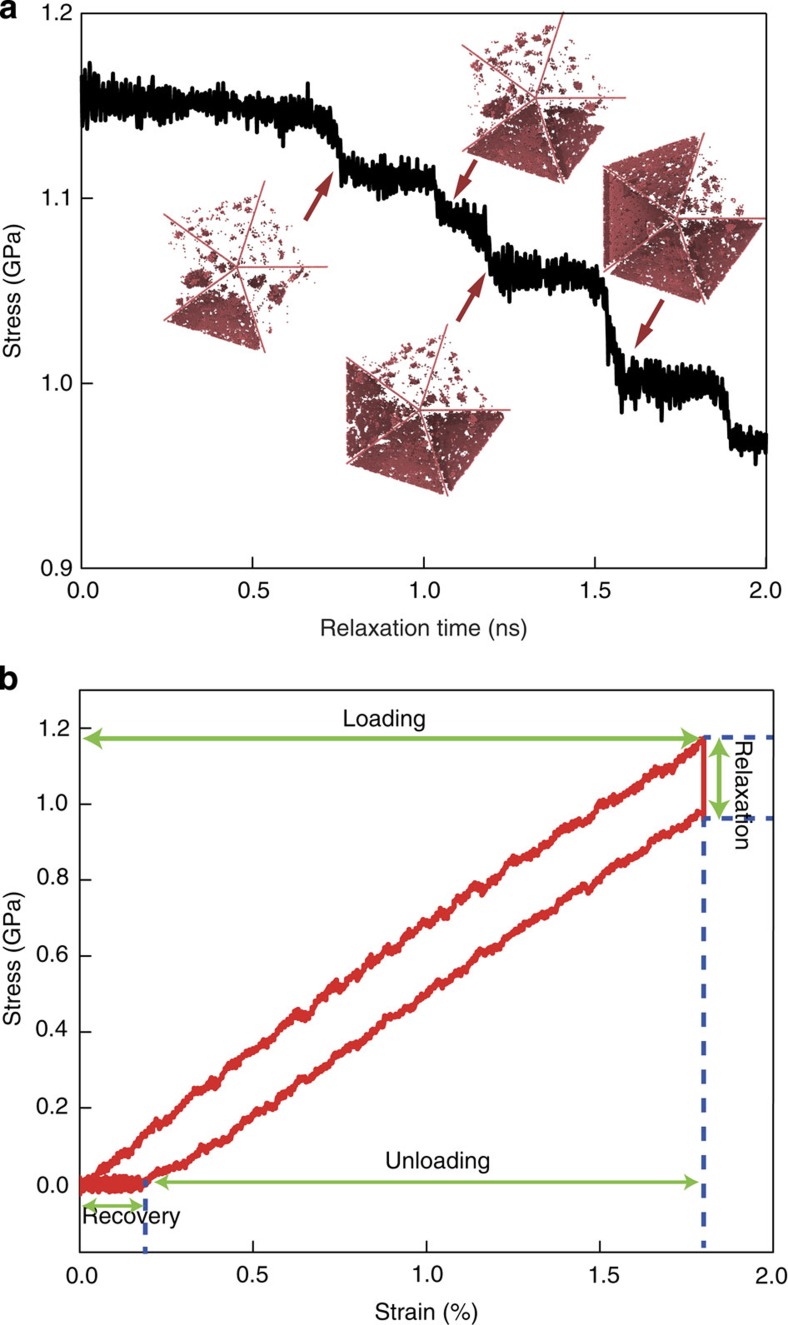
Simulated stress relaxation and strain recovery in penta-twinned NWs. (**a**) Stress relaxation of a 30 nm-diameter penta-twinned Ag NW. Each stress drop corresponds to a discrete event of dislocation nucleation shown in insets. (**b**) The stress–strain curve.

To reveal the underlying mechanisms behind the observed relaxation and recovery behaviours of penta-twinned Ag NWs, we performed a series of molecular dynamics (MD) simulations. Details of the simulation are supplied in Methods. It is known that stress relaxation is highly sensitive to the microstructures, particularly point defects^19^,^39^,^40^. Therefore, we introduced a population of vacancies with concentration from 0 to 2% in the simulated samples, based on experimental observations shown in Fig. 1d. To investigate stress relaxation behaviours, we first stretched the simulated samples to a strain of 1.8%, and then monitored stress relaxation while the applied strain was held fixed. The simulation results showed no stress relaxation during a simulation time period over tens of nanoseconds in a vacancy-free NW. However, with 1% vacancies introduced in the penta-twinned NWs, the stress gradually decreased from an initial stress of 1.15 GPa by about 200 MPa (that is, 17.4% of the initial stress) in 2 ns, as illustrated in Fig. 3a. The insets in Fig. 3a capture a sequence of partial dislocation nucleation events in the TB-separated nanograins. The partial dislocation nucleation events exhibit a one-to-one correspondence with discrete stress drops in the stress relaxation profile shown in Fig. 3a, indicating that the stress relaxation is a direct consequence of dislocation nucleation in the penta-twinned nanostructure.

**Figure 4:**
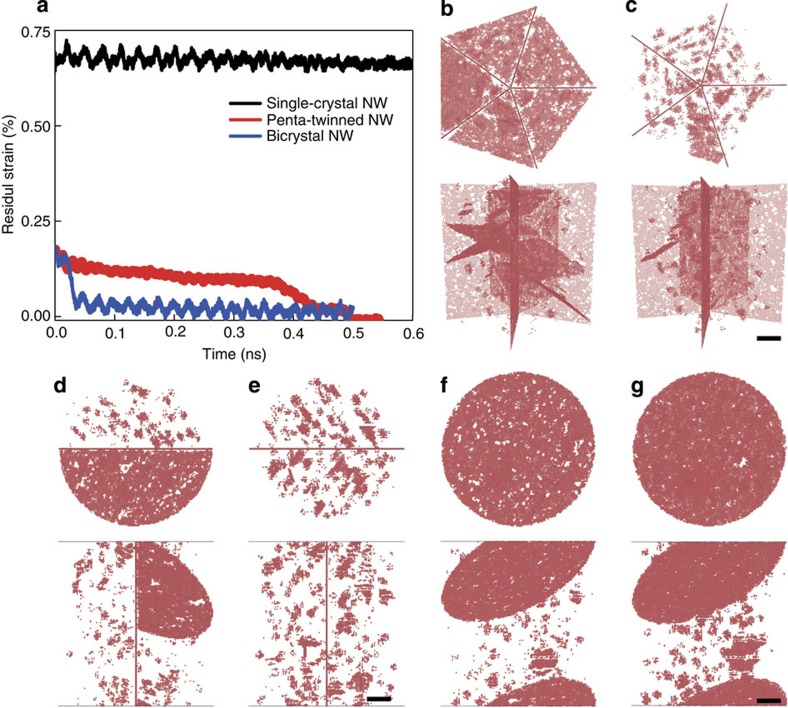
Recoverable plasticity and associated dislocation activities. (**a**) Time-dependent strain recovery due to the reverse motion of partial dislocations. (**b**,**c**) Penta-twinned NW; (**b**) during relaxation, dislocations are nucleated and confined by the penta-twinned nanostructure; (**c**) dislocations retract and disappear during recovery. (**d**,**e**) Bi-crystalline NW; (**d**) during relaxation, a dislocation is impeded by the TB; (**e**) the dislocation retracts and disappears during recovery. (**f**,**g**) Single-crystalline NW; (**f**) during relaxation, dislocations travel through the sample interior and escapes out of the free surface, leaving behind a permanent stacking fault; (**g**) after unloading, the stacking fault still resides in the sample interior, resulting in permanent plastic deformation. Only hexagonal close-packed atoms are visible in (**b**–**g**) for clarity, upper parts are viewed from NW axis and lower parts are side view of NWs. Scale bar, 5 nm.

Similar to experiments, after stress relaxation the simulated samples were subsequently unloaded to a stress-free state. When the applied stress came down to zero, there was still a 0.21% of strain remaining in the sample, as shown in Fig. 3b. Continuing relaxation of the sample under zero stress resulted in complete recovery of the residue strain after 0.5 ns (see Fig. 4a). This phenomenon is very similar to the experimental observations. Figure 4b,c illustrates two snapshots of the deformed sample during relaxation and recovery, respectively. During stress relaxation, partial dislocation loops were found to nucleate spontaneously from aggregated vacancy clusters near free surface and then expand through the grain interiors separated by the five TBs. Each discrete dislocation nucleation event leads to a visible stress drop in our simulation. During stress relaxation, it was observed that several dislocation loops moved and leaned against TBs as the stress level went down and some dislocation segments were absorbed by, while others stayed close to, the TBs. During subsequent strain recovery after unloading, partial dislocation loops were seen to retract from the fivefold TBs under zero applied stress, resulting in complete strain recovery. Details of such a process are shown in Supplementary Movie 5. It is known that there exists a repulsive force between TB and curved dislocation loop^41^,^42^. When the external stress is removed, the repulsive force from the TBs appears to induce reverse motion of dislocations by pushing the non-inserted segments as well as extracting the inserted segments from the TBs back towards where the dislocations were nucleated. Moreover, we have analysed the Peach–Koehler force exerted on the partial dislocations by the inhomogeneous intrinsic stress field of the fivefold twin and found that it will also act as a driving force for dislocation retraction during strain recovery (see Supplementary Note 1; Supplementary Figs 3–5). In addition, the intrinsic stress field of the fivefold twin has a certain gradient from the core to the free surface, which may assist vacancy diffusion.

While it is difficult to fully reveal the initial point defects in the NW samples, careful TEM examinations identified their presence near TBs (Fig. 1d). In accordance with this observation, we performed additional simulations with initial defects created in two neighbouring twin variants in the penta-twinned NWs. After equilibration, the overall vacancy concentration is measured to be around 0.1%. In such simulated samples with localized vacancy distribution near a TB, we also observed stress relaxation and plastic strain recovery (see Supplementary Movie 6).

## Discussion

In our simulations, the stress relaxation is significantly affected by vacancies in the NWs. When the vacancy concentration is increased by 0.5%, stress relaxation becomes more pronounced. Under a given initial stress, higher vacancy concentration leads to more dislocation nucleation events and larger stress drops (see Supplementary Fig. 6). To further understand how vacancy facilitates dislocation nucleation, we computed the generalized planar fault curves (essentially reflecting plastic deformation map) of Ag under different vacancy concentrations (see Supplementary Fig. 7). The results indicate that the vacancy reduces the energy barrier associated with the nucleation of leading partial dislocations in Ag. The relevant details are given in Supplementary Note 2. Remarkably, the ratio of unstable stacking fault energy to stacking fault energy *γ*_sf_/*γ*_usf_ decreases as vacancy concentration rises (see Supplementary Fig. 7). On the basis of previous studies^43^,^44^, such a phenomenon implies that full dislocations are getting harder to nucleate and the slip of energetically more favourable leading partials becomes a controlling plastic deformation mechanism. In addition, we performed a simulation for the uniaxial tension of a simple Ag block with (001) free surfaces to examine the influence of vacancy concentration on dislocation nucleation (see Supplementary Fig. 8). It is found that the first partial dislocation nucleates from the free surface at 4.15% strain in a perfect block, while this critical strain drops to 2.50% at vacancy concentration of 1%. These results indicate that the presence and aggregation of vacancies can promote the nucleation as well as operation of leading partial dislocations as a dominating deformation mechanism.

Previous studies^25^,^26^,^27^,^28^,^29^ showed that plastic strain recovery in thin films can be attributed to dislocation- and GB-mediated processes. In penta-twinned NWs, there are no regular GBs and the TBs have low energy, high symmetry, coherent atomic structure and one side geometrically constrained in the centre of the NWs. In this nanostructure, the sliding and diffusion processes should be suppressed to a large extent. Therefore, the controlling mechanism for the observed strain recovery behaviour can only be attributed to TB-dislocation interactions. The fivefold TBs separate the NW into five nanograins, which confine the length of dislocation segments as well as the mean free path of vacancy diffusion. More importantly, TBs are effective obstacles for dislocations and produce a repulsive force to drive the reverse motion of dislocations during unloading. In addition to these effects, the inhomogeneous stress field generated by the fivefold twin may also benefit vacancy diffusion and reversible dislocation motion.

To further demonstrate the unique role of TBs in the recovery behaviour of NWs, we performed two additional simulations for a single-crystalline NW and a bi-crystalline NW with a single TB in the middle—a mono-twinned NW. To facilitate dislocation nucleation in simulation, these two samples have the same vacancy concentration of 1.0%. Repeating the simulations as before but relaxing the two NWs from an initial strain of 2.0%, we observed nearly full plastic strain recovery in the mono-twinned NW, but not in the single-crystalline NW, as illustrated in Fig. 4a. In the mono-twinned NW, the TB effectively blocks the motion of leading partials, preventing them from escaping out of the sample (see Fig. 4d,e) and allowing them to retract back from the TB under the residual internal stress field on unloading, leading to plastic strain recovery, albeit not as complete as the penta-twinned case. In contrast, in the single-crystalline NW, dislocations nucleated from the free surface, travelled across the NW and eventually escaped out of the sample, leaving a permanent surface step (see Supplementary Movies 7 and 8). In this case, the dislocation motion, and the associated deformation, becomes irreversible during subsequent unloading. Besides the twinned NW, we have also investigated bi-crystalline NWs with different types of regular GBs (such as low-angle/high-angle tilt GBs and mixed GB). It is found that the tilt and mixed GBs either failed to block the dislocation or trapped the dislocation, resulting in no strain recovery (see Supplementary Note 3; Supplementary Fig. 9; Supplementary Movies 9 and 10). These studies further demonstrate that the TBs play an essential role in the reverse motion of partial dislocations, which lead to the observed plastic strain recovery in the penta-twinned Ag NWs. It is noted that, in penta-twinned NWs, fivefold TBs divide the NW interior into five nanograins, which can prevent the escape of dislocations from free surface and facilitate dislocation retraction more effectively compared with a single TB in bi-crystalline NWs. In addition, the intrinsic residual stress field induced by the fivefold twin also favours dislocation retraction on unloading.

We have also investigated the influence of sample diameter on the observed deformation behaviour in penta-twinned Ag NWs (see Supplementary Figs 10 and 11; Supplementary Note 4), with results showing that the characteristic time scale (that is, relaxation time) for the observed behaviour depends on the sample diameter (see Supplementary Tables 1 and 2). As the sample diameter decreases, the relaxation time increases, in agreement with both our experiments and simulations. Such size dependence on the relaxation time is essentially attributed to the size effect of dislocation nucleation. The relaxation time should depend on the activation energy and stress for dislocation nucleation. Under the same temperature and strain rate, the critical stress for dislocation nucleation from free surfaces (in the case of NWs) was found to be inversely proportional to the NW diameter. Therefore, stress relaxation should be less prominent in smaller samples than in larger samples. A more detailed study on the size dependence of the relaxation and recovery behaviours will be performed in the future.

Plastic deformation in metals generally consists of two components: an athermal part and a thermal part. The former, driven by the long-range internal stress, has a weak temperature dependence, while the latter accounts for thermally activated processes that are usually sensitive to both temperature and strain rate. In our experiments and simulations reported above, the NWs did not reach the athermal yield point during loading. It was during the relaxation step that the thermally activated nucleation of partial dislocations was observed.

To investigate whether the phenomenon of recoverable plasticity also occurs for similar samples beyond the athermal plastic yield point, we have performed additional MD simulations to investigate the loading–unloading behaviour of the penta-twinned Ag NWs under much larger strains. The NWs were loaded beyond the athermal yielding point such that partial dislocations are nucleated even without the relaxation step. In the first simulation, the sample was loaded to 4.5% strain, which is only slightly beyond the athermal yielding point (see Supplementary Fig. 12). In this case, a number of partial dislocations were nucleated during loading and then retracted back to their original nucleation sites during unloading, still resulting in full plastic strain recovery. During this process, none of the partial dislocations cut across the TBs. In the second simulation, the sample was loaded to about 10% strain, corresponding to very large plastic deformation before unloading (see Supplementary Fig. 13). There was only about 1% strain recovered during unloading. In this case, the applied stress was large enough to allow dislocations to cut across the TBs, and multiple slip systems were activated, leading to reactions and entanglement between dislocations from different slip systems. As a result, more complex and disordered dislocation structures prevented full strain recovery and only 1% strain was observed to recover, reminiscent of the classical Bauschinger’s effect.

In summary, we have discovered an unusual dislocation-based stress relaxation and strain recovery behaviour in penta-twinned Ag NWs via *in situ* SEM/TEM tensile testing, TEM microstructural characterizations and large-scale MD simulations. This behaviour consists of two facets: stress relaxation on loading and complete plastic strain recovery on unloading. Our studies indicate that stress relaxation originates from the nucleation of leading partial dislocations assisted by vacancy diffusion, while the complete strain recovery is due to the reverse motion of partial dislocations driven by the repulsive force from the TBs and the intrinsic stress field due to the fivefold twin. The simulations demonstrate that the TBs play a crucial role in the observed phenomenon in penta-twinned NWs, as bi-crystalline NWs with a single TB exhibit a similar behaviour, while single-crystalline NWs do not. It is noted that diffusion and sliding processes associated with GBs should be suppressed in penta-twinned NWs. Therefore, in contrast to previous studies in nanocrystalline metals due to a cooperation of dislocation- and GB-mediated processes, the present study demonstrates a new type of stress relaxation and full plastic strain recovery induced by TB-dislocation interactions. Our study opens up a promising prospect of designing 1D nanostructures with time-dependent strain recovery capabilities.

## Methods

### Sample synthesis and characterization

Penta-twinned Ag NWs were synthesized by reducing AgNO_3_ with ethylene glycol in the presence of polyvinyl pyrrolidone. More details of the NW synthesis process are provided elsewhere^45^. The solution of Ag NWs was diluted with deionized water and then purified by centrifugation. Single-crystalline Ag NWs were synthesized by physical vapour deposition inside a molecular beam epitaxy system under ultrahigh vacuum condition and substrate temperature of 700 °C. More details of the NW synthesis process are provided elsewhere^4^.

Conventional TEM observations were performed on JEOL 2010F operated at 200 kV. Atomic-resolution high-angle annular dark-field scanning TEM imaging was performed on a probe-corrected FEI Titan G^2^ 60–300 kV S/TEM equipped with an extreme field emission gun (X-FEG) electron source operated at 200 kV.

Cross-section TEM samples were prepared by embedding Ag NWs into Gatan G1 epoxy with a φ3 Cu tube, cutting the specimen discs with a thickness of ~0.5 mm, mechanically polishing with Allied Multiprep System and finally ion milling the sample via Gatan 791 PIPS while cooling with liquid nitrogen.

### *In situ* SEM/TEM mechanical testing

*In situ* mechanical testing inside a SEM or TEM was carried out using a MEMS-based material-testing system, which consists of a thermal actuator, a capacitive load sensor and a gap in between for mounting samples. The displacement markers are deposited using electron beam-induced deposition of carbon. Ag NWs were mounted on the testing stage using a nanomanipulator (Klocke Nanotechnik, Germany) inside a FEI Nova 600 dual beam and clamped by carbon deposition. The loading and unloading strain rates for *in situ* SEM tensile testing were ~0.1% s^−1^. During loading, the actuator was loaded incrementally to a prescribed displacement. During relaxation, the actuator displacement was held constant, and the specimen relaxed as a function of time. More specifically, the load on the specimen decreased and the specimen elongation increased at the same time. During recovery, the actuator was turned off and retracted to the original position.

*In situ* TEM mechanical testing was performed on JEOL 2010F operated at 200 kV. The loading and unloading strain rates were ~0.005% s^−1^. Low-magnification images were recorded at a fixed condense current to minimize the focus change. Real-time videos were recorded at a speed of three frames per second.

### Atomistic simulations

Large-scale MD simulations were performed using the software package LAMMPS^46^. Three kinds of NW samples are generated: a penta-twinned NW with a fivefold twin, a bi-crystalline NW with a single coherent TB in the middle and a single-crystalline NW. Supplementary Figure 14 shows the atomic configurations of the three kinds of simulated samples. Each sample is 30 nm in diameter and 30 nm in length and contains about 1.3 million atoms. The embedded atom method potential for Ag^47^ is used to describe the interatomic interactions. The vacancies are introduced by randomly removing atoms out of the samples.

The samples are initially relaxed and equilibrated at an elevated temperature of 800 K (about 65% of the melting point of Ag) for 80 ps using the Nosé–Hoover thermostat and barostat^48^. Periodic boundary condition is imposed along the axial direction (that is, the loading direction <110>) of all simulated samples. The samples are first stretched to a certain strain of 1.8 or 2% at a constant strain rate of 10^8^ s^−1^ under NVT ensemble (canonical ensemble), and then relaxed for several nanoseconds under NVT ensemble while holding the strain fixed. In the relaxation process, we monitor the variation of the axial stress by averaging the virial stresses^49^ over all atoms in the samples. Note that we have used an elevated temperature of 800 K in our MD simulations to accelerate the thermally activated processes of vacancy diffusion and the associated dislocation nucleation within the time scale of MD simulations. To examine the reversibility of deformation (related to the reverse motion of dislocations) and measure the residual strain, we unload the elongated samples at a strain rate of −10^8^ s^−1^ until the axial stress in the simulated samples approaches zero. Subsequently, the samples are further relaxed at the equilibrium state while the axial stress is kept zero by a Nosé–Hoover barostat. The residual strain is calculated by comparing the initial and final lengths of the samples. Throughout the simulations, the temperature is kept constant at 800 K by the Nosé–Hoover thermostat. The loading and unloading processes are designed to mimic the real experimental testing apart from much higher strain rates.

To identify defects during deformation of the samples, we paint atoms with different crystalline order in different colours using a common neighbour analysis. The green-coloured atoms stand for atoms with face-centred cubic symmetry, the red ones those with hexagonal close-packed symmetry and the grey ones those at dislocation cores, surfaces and point defects.

## Author contributions

Y.Z. and H.G. conceived the project and designed the experiments and simulations; Q.Q., G.C. and T.-H.C. performed the *in situ* experiments; S.Y. and X.L. performed the atomistic simulations; G.R. provided the single-crystalline Ag NW samples; all authors analysed the data, discussed the results and wrote the manuscript.

## Additional information

**How to cite this article:** Qin, Q. *et al.* Recoverable plasticity in penta-twinned metallic nanowires governed by dislocation nucleation and retraction. *Nat. Commun.* 6:5983 doi: 10.1038/ncomms6983 (2015).

## Supplementary Material

Supplementary Information Supplementary Figures 1-14, Supplementary Tables 1-2, Supplementary Notes 1-4 and Supplementary References

Supplementary Movie 1 In-situ SEM observations of loading, relaxation, unloading and strain recovery of penta-twinned Ag NW.

Supplementary Movie 2 In-situ TEM observations of dislocation nucleation and propagation in a penta-twinned Ag NW at the relaxation stage. The green box shows the viewing area in the rest of the movie. No dislocations were observed to start with. At relaxation of ~ 5 minutes, dislocations (arrowed) were observed in the boxed area (note that we did not know where dislocations would nucleate a priori). The dislocations remain at the same spot for the rest of the relaxation step.

Supplementary Movie 3 In-situ TEM observations of dislocation annihilation in a penta-twinned Ag NW at the recovery stage. The arrow marks where the dislocations were. Note that we did not mark the arrow at every frame.

Supplementary Movie 4 In-situ SEM observations of loading and unloading in single-crystalline Ag NW.

Supplementary Movie 5 MD simulation of relaxation, unloading and recovery processes of a penta-twinned Ag NW. During relaxation, partial dislocations are nucleated in each grain and confined by the five-fold twin. During unloading and recovery, these same dislocations retract back to where they are nucleated, resulting in fully reversible plastic deformation.

Supplementary Movie 6 MD simulation of relaxation, unloading and recovery processes of a Ag NW with pre-existing defects. Vacancies are introduced in two of the five grains, with total vacancy concentration kept at about 0.1%. During relaxation, partial dislocations are nucleated in the defective grains and confined by the adjacent twin boundaries. The retraction of these partial dislocations during unloading and recovery leads to strain recovery.

Supplementary Movie 7 MD simulation of relaxation, unloading and recovery processes of a single-crystalline Ag NW. During relaxation, partial dislocations nucleate at the free surface, travel through the NW and escape out of the sample, leaving behind stacking faults across the whole section of the NW. During unloading and recovery, the stacking fault stays in the sample and the resulting plastic deformation becomes irreversible.

Supplementary Movie 8 MD simulation of relaxation, unloading and recovery processes of a bi-crystalline Ag NW with a single twin boundary in the middle. During relaxation, partial dislocations nucleated from the free surface are blocked by the twin boundary. The retraction of these dislocations upon unloading and recovery leads to reversible plastic deformation.

Supplementary Movie 9 MD simulation of relaxation, unloading and recovery processes of a bi-crystalline Ag NW with a 7° tilt grain boundary in the middle. During relaxation, partial dislocations nucleated from the free surface easily traverse through the tilt grain boundary and escape at the opposite surface of the sample, leaving behind permanent stacking faults in the NW. During unloading and recovery, the stacking faults stay in the sample and the resulting plastic deformation becomes irreversible.

Supplementary Movie 10 MD simulation of relaxation, unloading and recovery processes of a bi-crystalline Ag NW with a 45° tilt grain boundary in the middle. During relaxation, partial dislocations nucleated from the free surface are blocked by the tilt grain boundary in the middle of the NW. During unloading and recovery, the stacking fault stays in the sample and the resulting plastic deformation becomes irreversible.
