# Supplementary material for: Recoverable plasticity in penta-twinned metallic nanowires governed by dislocation nucleation and retraction
Source: Nat Commun. 2015 Jan 13;6:5983. doi: 10.1038/ncomms6983 (PMC4308715; doi:10.1038/ncomms6983)
Supplement: Supplementary Information — Supplementary Figures 1-14, Supplementary Tables 1-2, Supplementary Notes 1-4 and Supplementary References [file ncomms6983-s1.pdf]

Supplementary information

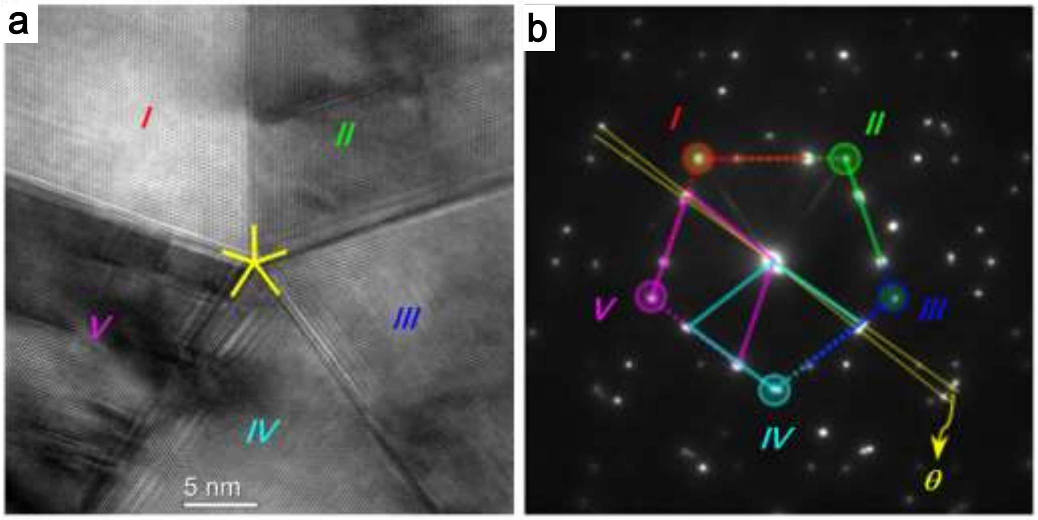

Supplementary Figure 1 | HRTEM image and SAED pattern of the cross-sectional atomic structure of a penta-twinned NW. (**a**) HRTEM image showing a high density of stacking faults distributed around the boundary between grains IV and V. The five twin variants are numbered from I to V, corresponding to the marked {002} diffraction spots in Supplementary Fig. 1b. Note that the white or black fringes are caused by unfocused imaging conditions. The boundary between twin variants IV and V is not an ideal TB. It has a small included angle marked as angle θ in the SAED pattern (Supplementary Fig. 1b), which is measured to be ~4˚. (**b**) SAED pattern of a cross-sectional penta-twinned Ag NW sample. Stacking faults along the boundary between twin variants IV and V can be clearly seen in (a). The yellow star in (a) indicates the cross-sectional center of the sample. Based on our TEM and HRTEM observations, it is a universal phenomenon that three or four of the boundaries keep perfect twin relationships while the rest two or one has a small included angle from 1.5 to 5.5˚. Collectively they compensate the mismatched angle (7.4˚) to form a "five-fold" twinning structure.

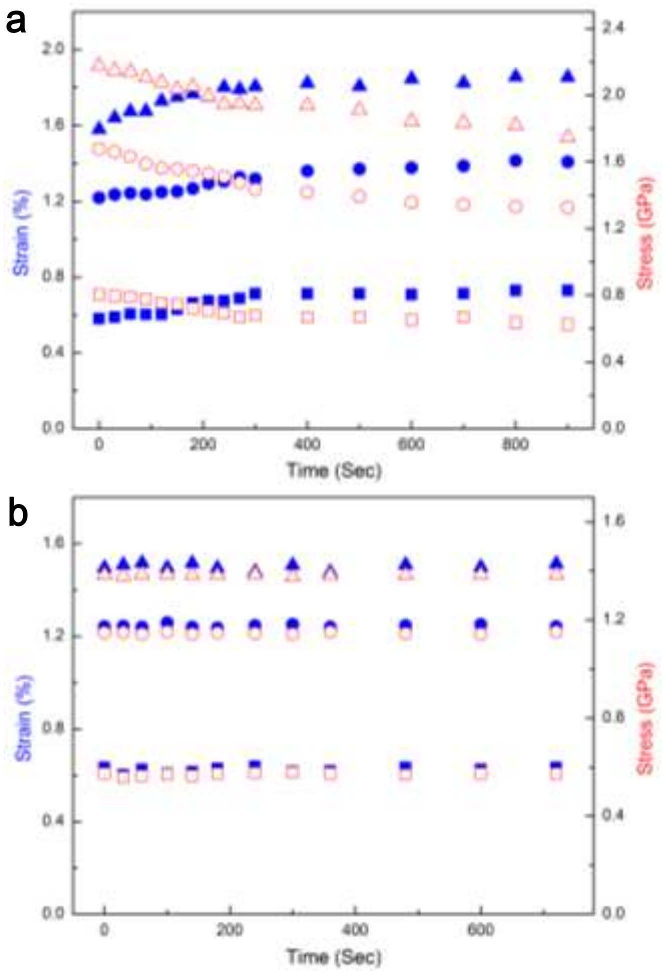

Supplementary Figure 2 | Relaxation curves of additional penta-twinned and single-crystalline Ag NWs (**a**) penta-twinned Ag NW with a diameter of 48 nm and (**b**) single-crystalline Ag NW with a diameter of 130 nm. Solid and open symbols correspond to the strain-time and stress-time relationships, respectively. Square, circle and triangle symbols correspond to 1st, 2nd and 3rd relaxation at different strains, respectively.

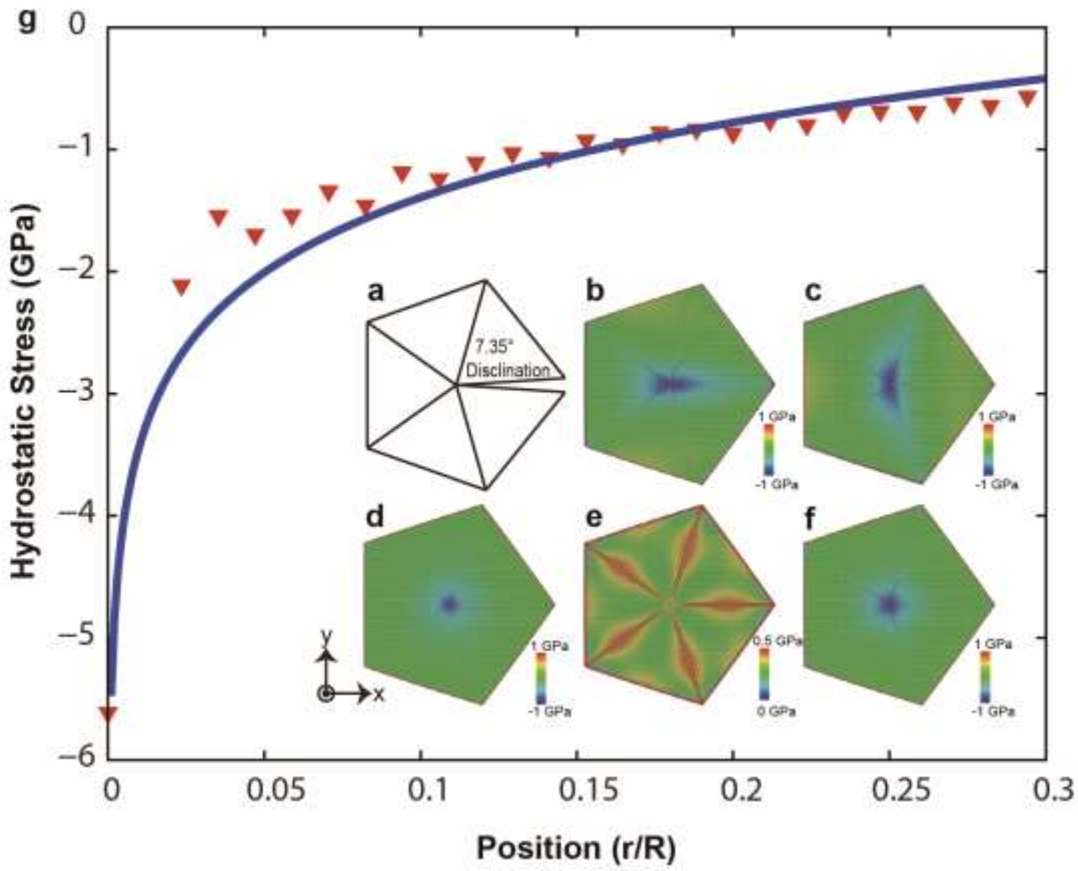

Supplementary Figure 3 | Intrinsic stress field in the penta-twinned nanostructure. (**a**) Sketch of the disclination for the penta-twinned structure. (**b-f**) Contours of normal (σ_{xx}, σ_{yy} and σ_{zz}), von Mises and hydrostatic stresses from atomistic simulations, respectively. (**g**) Hydrostatic stress as a function of *r*/*R*, *r* being the radial distance and *R* the radius of NW. The blue solid curve represents the exact solution from disclination theory, while the red triangles are the results from atomistic simulation.

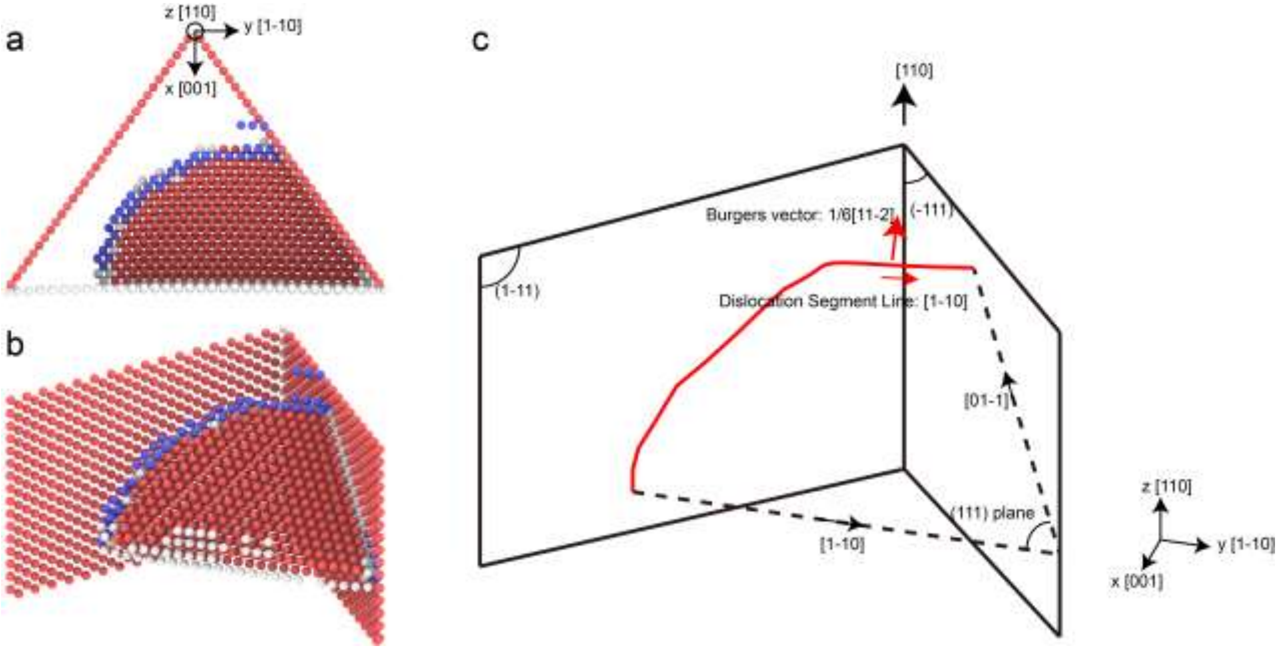

Supplementary Figure 4 | Nucleation and propagation of a Shockley partial dislocation in one grain of the penta-twinned nanowire. (**a-b**) Atomic structures of the partial dislocation. (**c**) Schematic illustration of the dislocation gliding on its slip plane.

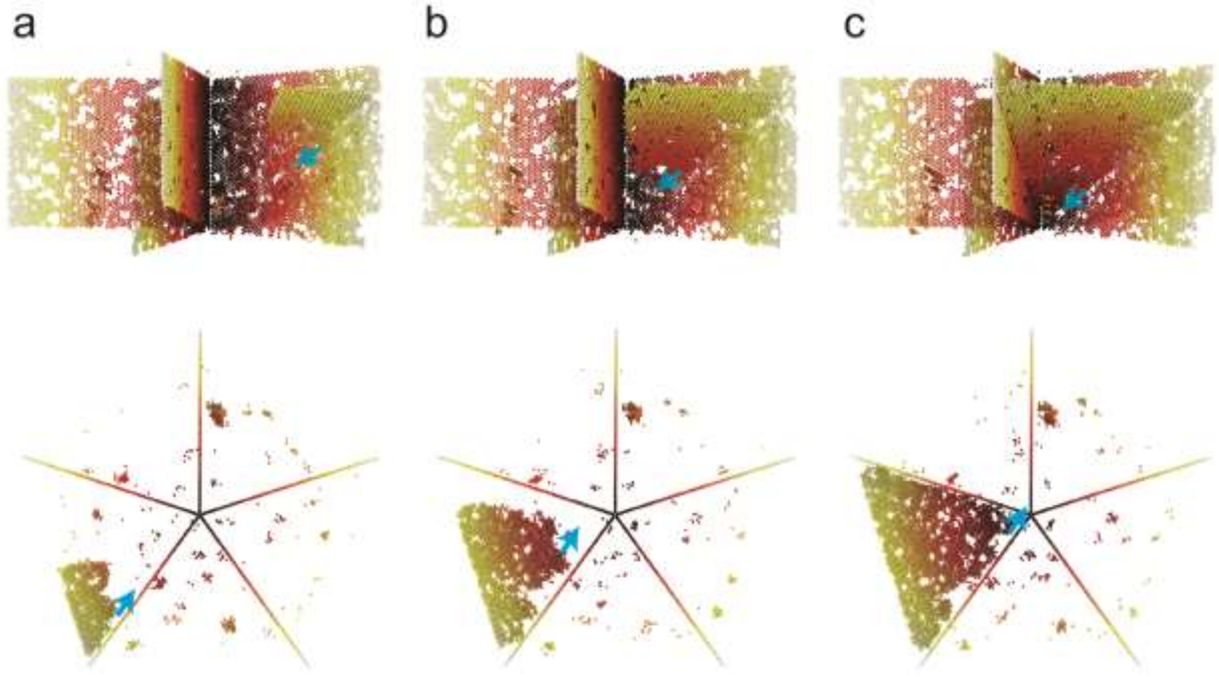

Supplementary Figure 5 | A sequence of snapshots of nucleation and propagation of partial dislocations in the penta-twinned nanowire. A Shockley partial dislocation nucleates from the intersection between a TB and the free surface and then glides on its slip plane. The slip direction of the dislocation is indicated by the blue arrow. For clarity, atoms in defects are colored according to the distances from atoms to the center.

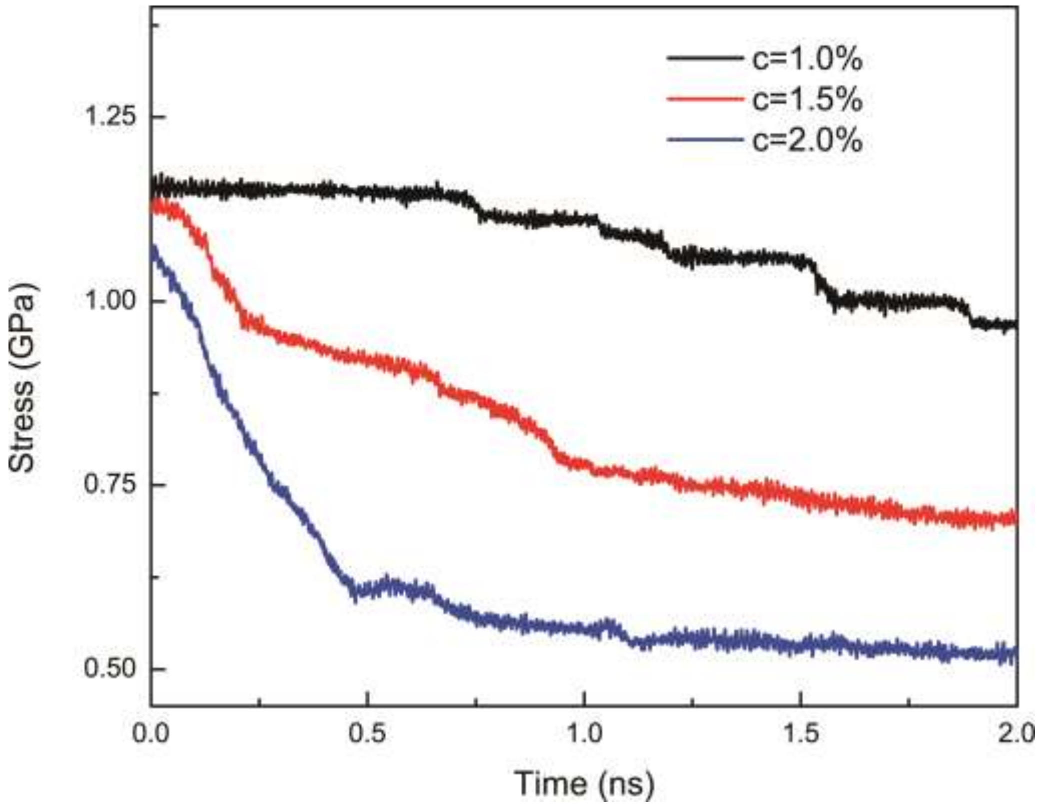

Supplementary Figure 6 | Stress relaxation of a 30 nm-diameter penta-twinned Ag NW at different levels of vacancy concentration c. The figure shows that higher vacancy concentrations lead to more pronounced stress relaxation and smaller characteristic relaxation time which indicate that stress relaxation in penta-twinned NW is closely associated with vacancy concentration.

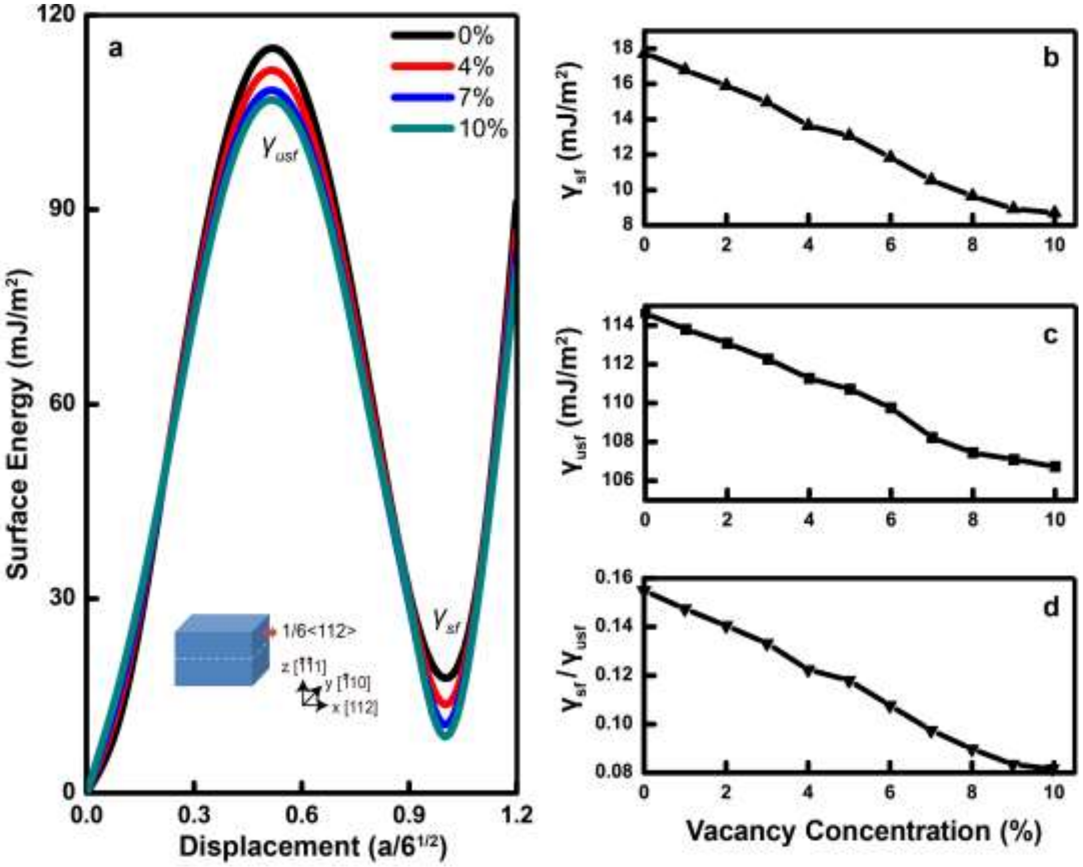

Supplementary Figure 7 | Influence of vacancy concentration on stacking fault energies. (**a**) Generalized planar fault curves for different vacancy concentrations. (**b-d**) Variations of γ_{sf}, γ_{usf} and γ_{sf}/γ_{usf} as functions of the vacancy concentration.

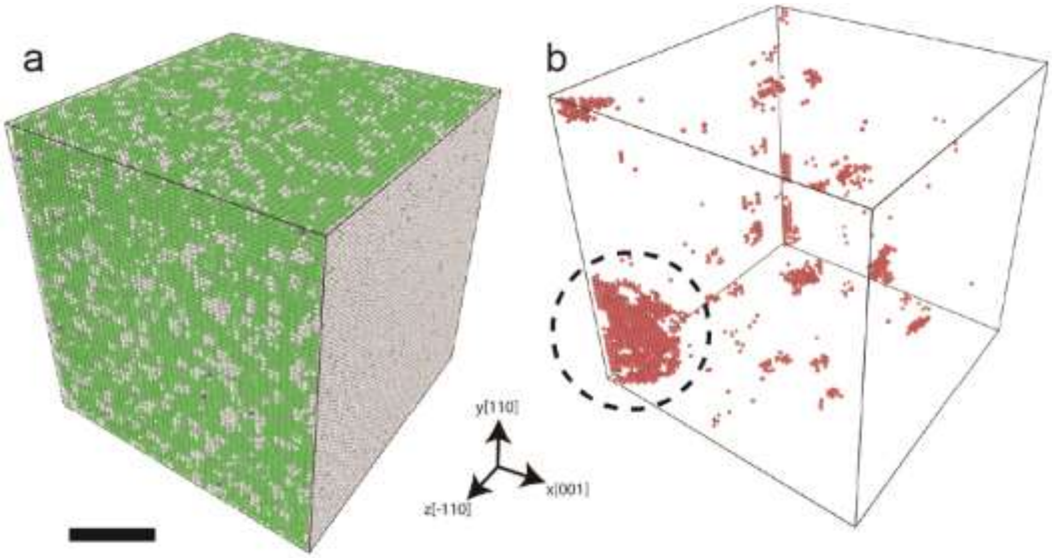

Supplementary Figure 8 | Surface dislocation nucleation in a twin-free Ag block with 1% vacancy. (**a**) Atomic configuration. Scale bar, 5nm. (**b**) Dislocation nucleates from free surface at strain of 2.50%, which is smaller than the corresponding critical strain of 4.15% required for dislocation nucleation in a vacancy-free sample (only HCP atoms are shown).

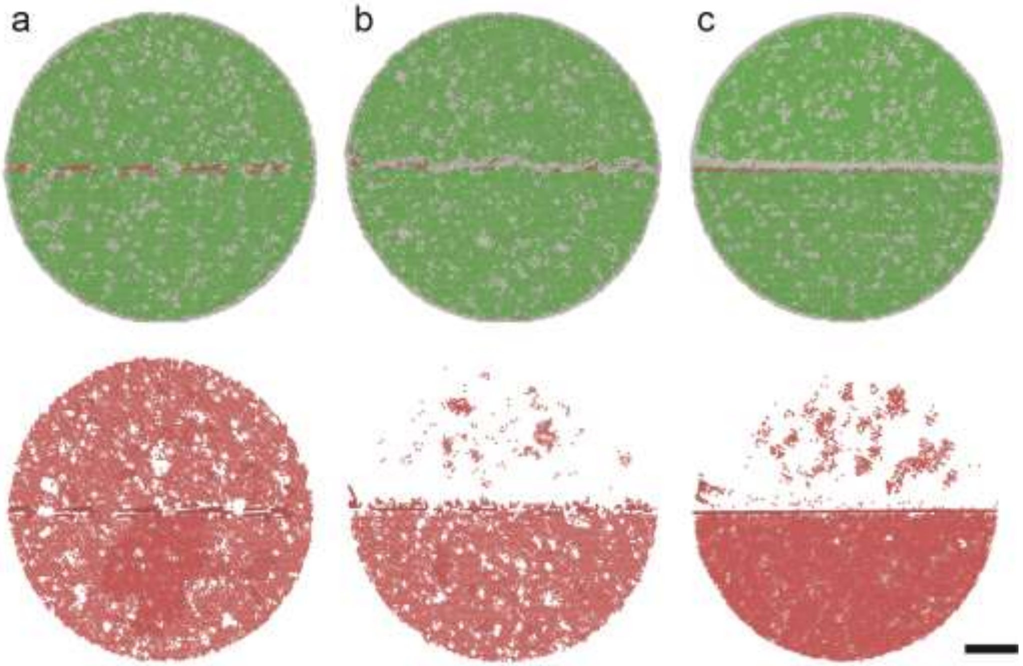

Supplementary Figure 9 | Cross-sectional view of bi-crystalline NWs with different types of grain boundaries before and after relaxation. The upper figures show the cross-section atomic configurations of NWs before relaxation, and the lower ones display their configurations after relaxation (only HCP atoms are shown). (**a**) In the case of a low angle 7° tilt grain boundary, dislocations can easily traverse through the grain boundary and escape out of the sample, leaving behind stacking faults in the NW. (**b**) In the case of a 45° tilt grain boundary, dislocations are blocked by the grain boundary but they fail to retract upon unloading. (**c**) In the case of a tilt-twist mixed grain boundary, dislocations are also impeded by the grain boundary, but they also fail to retract upon unloading. In all these cases, no strain recovery is observed. Scale bar, 5nm.

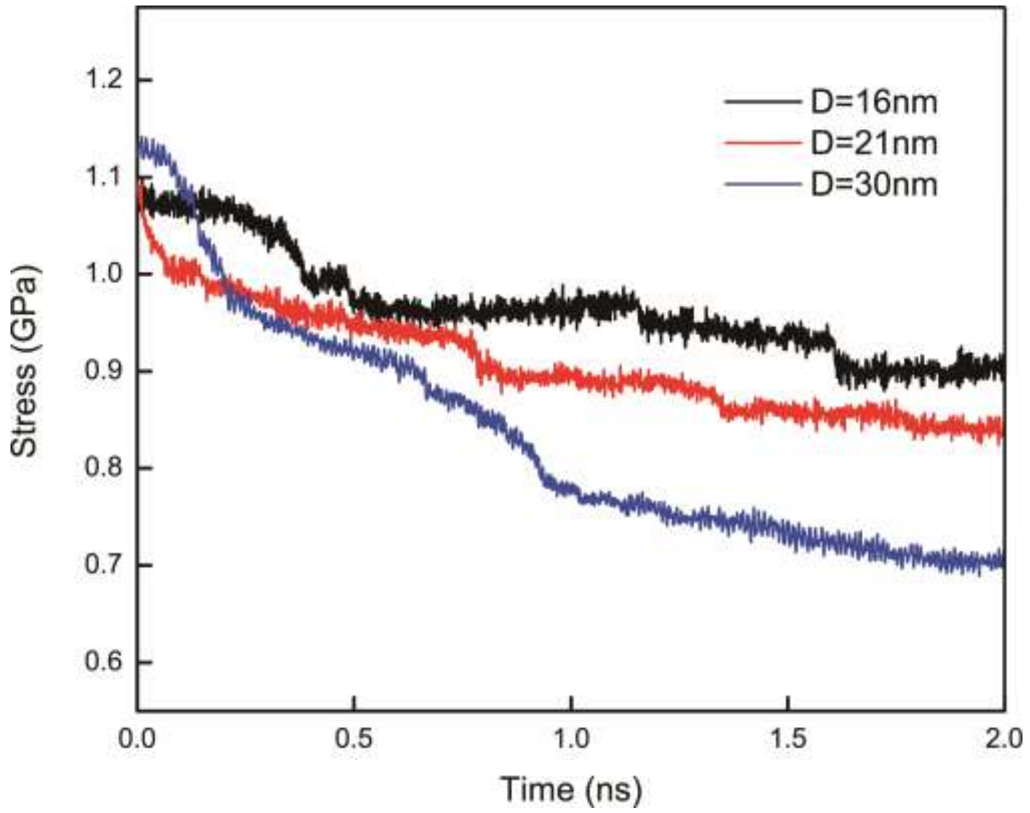

Supplementary Figure 10 | Stress relaxation in penta-twinned NWs with different diameters under the same vacancy concentration of 1.5%. In all these cases, the initial strain for relaxation is about 1.8%. Experimental study[5] has shown that the yield strain and stress of penta-twinned Ag NWs depend strongly on the extrinsic dimension of NWs. As the diameter of NWs decreases, both yield strain and stress related to incipient plasticity (corresponding to discrete dislocation nucleation from surface) increase dramatically. This means that dislocation nucleation is more difficult in smaller samples than in larger ones. Therefore, stress relaxation should be less prominent in smaller samples than in larger samples, as shown in this figure. In Supplementary Note 4, we will further address the influence of sample diameter on the characteristic time scale for stress relaxation.

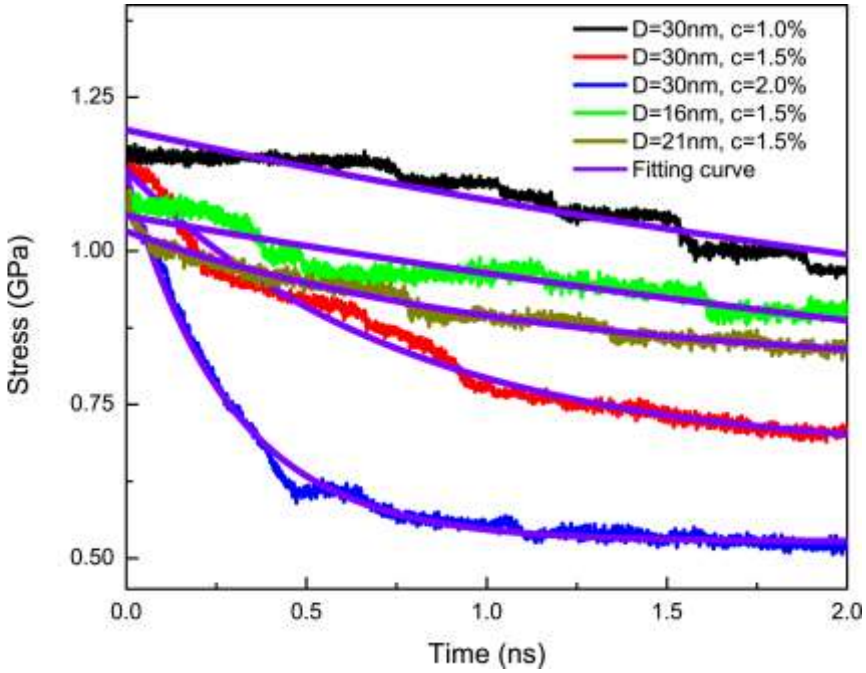

Supplementary Figure 11 | Simulated and fitting curves of stress relaxation (c is vacancy concentration).

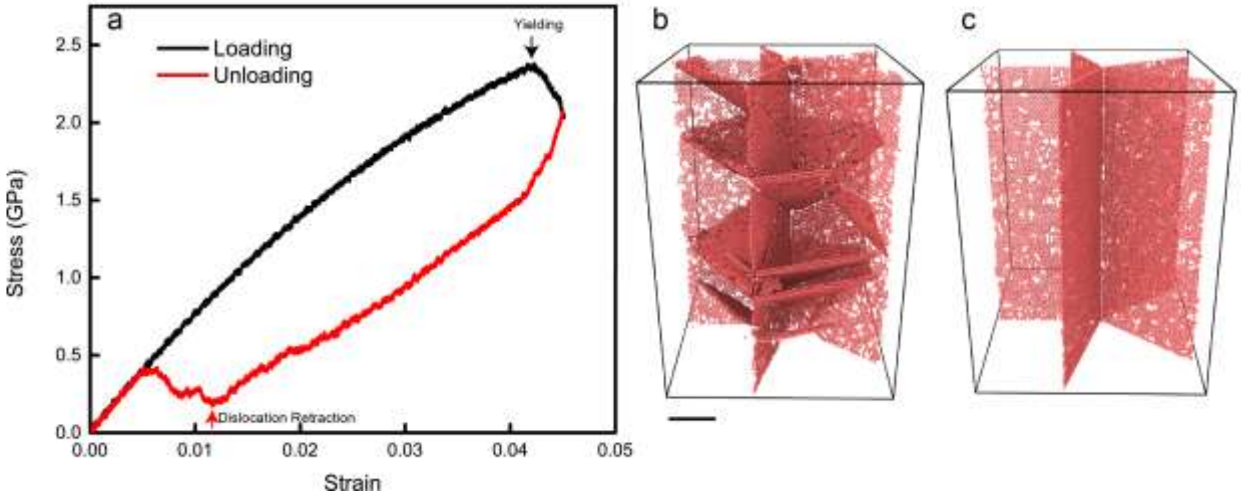

Supplementary Figure 12 | MD simulations of a penta-twinned nanowire loaded to 4.5% strain, which is slightly beyond the athermal yielding point, and then unloaded. To investigate whether the phenomenon of recoverable plasticity also occurs in a penta-twinned NW beyond the athermal plastic yield point, we performed additional MD simulations. The simulation sample is 20 nm in diameter and 30 nm in length, and contains about 0.6 million atoms. The simulation methods are the same as those in the previous simulations (where the relaxation and unloading start below the athermal yielding point). Since the sample was loaded beyond the athermal yielding point, no relaxation step was needed to nucleate partial dislocations. Therefore, we skipped the relaxation step and no initial vacancies were introduced in these simulations. (**a**) Stress-strain curve in one loading-unloading cycle showing fully recoverable plasticity. Two critical points are indicated by arrows. At the first point (i.e. the yielding point), a number of dislocations are nucleated and start to slip, leading to an apparent stress drop. At the second critical point, dislocations start to move backwards during unloading, resulting in subsequent full plastic strain recovery. (**b**) Atomic configuration of the nanowire at 4.5% strain which shows that a number of partial dislocations are nucleated, slip and interact with twin boundaries during loading, so that many stacking faults associated with partial dislocations are generated in the nanowire. (**c**) Atomic configuration of the nanowire after unloading to zero-stress. Upon unloading, all these partial dislocations retract back to their original nucleation sites, and the stacking faults disappear. For clarity, only HCP atoms are shown in (b) and (c). During this process, none of the partial dislocations are transferred across the twin boundaries. Scale bar, 5nm.

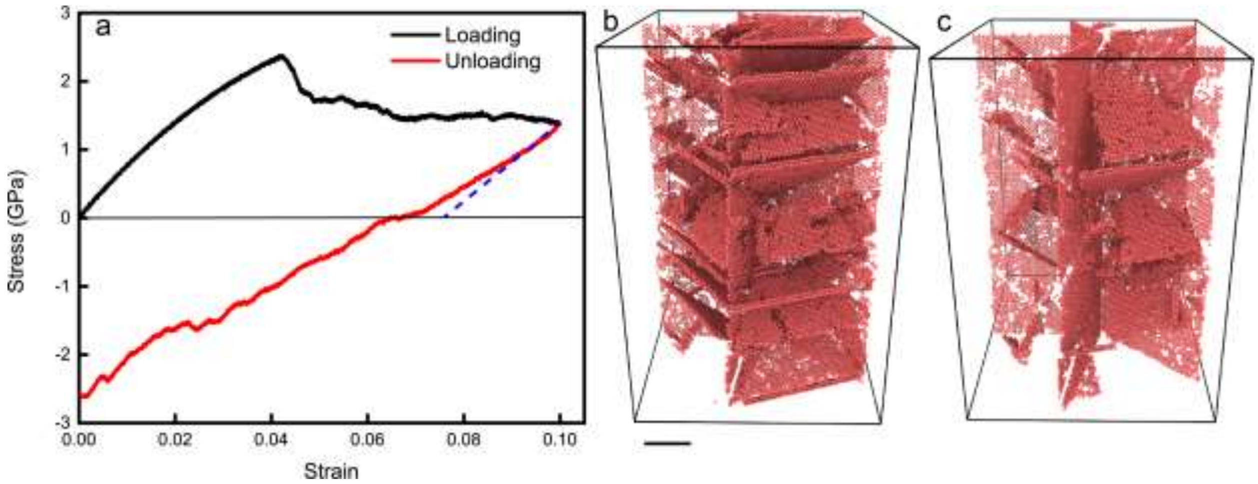

Supplementary Figure 13 | MD simulations of a penta-twinned nanowire loaded to 10% strain and then unloaded which corresponding to relatively large plastic deformation, before unloading. (**a**) Stress-strain curves showing partial recovery of plastic strain where a blue dashed line with the same slop as the initial elastic loading is used to identify the recovered strain. There is about 1% of strain recovered during unloading, which is only a small part of the total plastic strain. (**b**) Atomic configuration of the nanowire at 10% strain. Some dislocations have cut across the twin boundaries (due to the large applied stress in the sample) during loading, decomposing into penetrating segments entering the neighboring twin and some residual ones staying on the twin boundary. Also, as the plastic strain increases, multiple slip systems are activated, leading to reactions and entanglement between dislocations from different slip systems. In this case, we observed more permanent plastic deformation, and more complex and disordered dislocation structures that prevented full strain recovery. (**c**) Atomic configuration of the nanowire after unloading to zero-stress. Only a few dislocations are able to retract, leading to recovery of a small portion of the total plastic strain. Most dislocations react with each other or twin boundaries, leaving complex debris patterns. For clarity, only HCP atoms are shown in (b) and (c). Scale bar, 5nm.

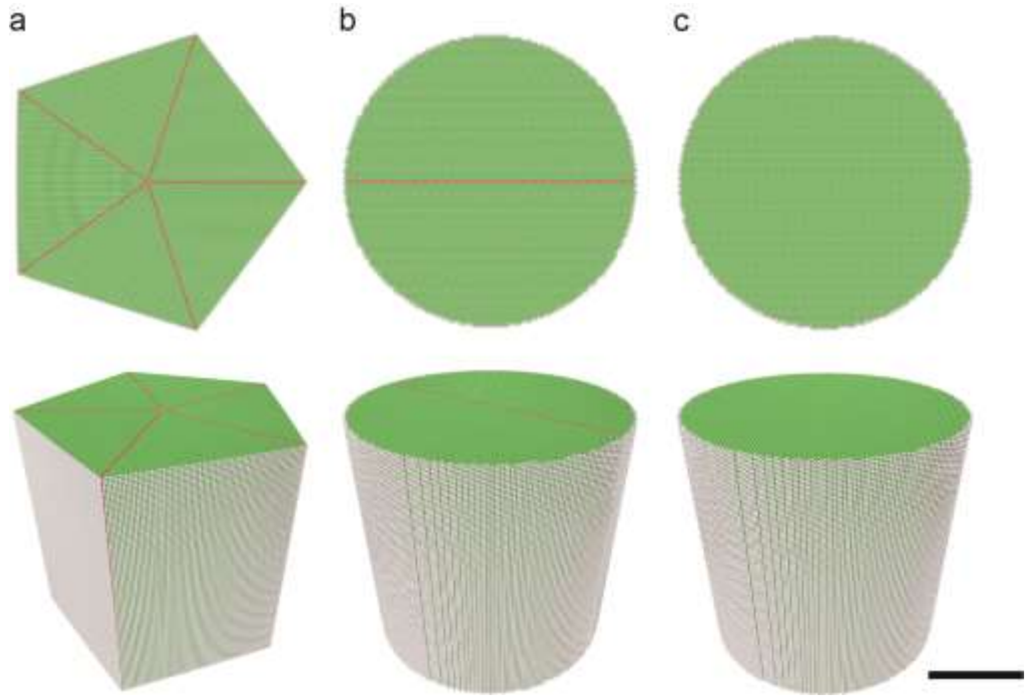

Supplementary Figure 14 | Atomic configurations of three kinds of NW samples. (**a**) penta-twinned, (**b**) mono-twinned bi-crystalline, and (**c**) single-crystalline NWs. The upper parts are viewed from NWs axis and the lower parts are side views of NWs. Scale bar, 10nm.

**Sample dimension *D* (nm)**	**Relaxation time τ_ε (s)**
112	94
	137
	108
104	187
	141
	166
48	191
	179
	185

Supplementary Table 1 | Fitting parameters for experimental cases at *T*=300 K.

**Sample dimension *D* (nm)**	**Vacancy concentration (%)**	**Relaxation time τ_ε (ns)**
30	1.0	3.96
	1.5	0.76
	2.0	0.29
21	1.5	1.09
16	1.5	5.12

Supplementary Table 2 | Fitting parameters for simulation cases at *T*=800 K (initial strain ε_0=1.8%).

Supplementary Note 1 | Intrinsic residual stress field of five-fold twin

For FCC structures, the angle between adjacent {111} planes is 70.53º. Actually, the five-fold twin structure can be regarded as a disclination with an angular deficiency of 7.35º (as shown in Supplementary Fig. 3a). Such a structure carries an intrinsic stress field due to the angular elastic misfit[1]. Supplementary Figures 3b-f show the internal normal stresses (σ_{xx}, σ_{yy} and σ_{zz}), von Mises stress and hydrostatic stress contours of a five-fold twin from atomistic simulations at a constant temperature of 1.5 K, respectively. The highest tensile stress occurs at the free surface, while the lowest compressive stress occurs at the core. The von Mises stress exhibits a quinquefoliate-like distribution, with local maximum on twin boundaries and local minimum at the bisecting line of two neighboring boundaries[1]. Such stress distribution is thought to be the origin of pronounced stiffening of smaller penta-twinned NWs[1]. In the region close to the core of the five-fold twin, the hydrostatic stress distribution at the atomic scale is in good agreement with theoretical predictions from the continuum disclination theory[2], as demonstrated in Supplementary Fig. 3g. Notably, the intrinsic stress field caused by the five-fold twin is significantly inhomogeneous, and thus could serve as a driving force to the reverse motion of dislocations responsible for strain recovery. Moreover, such stress field has a certain gradient from the core to the free surface, which may assist vacancy diffusion.

Supplementary Figure 4 shows a Shockley partial dislocation in one of the penta-twinned NW grains, and Supplementary Figure 5 captures a typical process of dislocation nucleation and propagation in the penta-twinned NW. We investigate the Peach-Koehler force exerted on the partial dislocation segment by the intrinsic stress field of the five-fold twin. As illustrated in the Supplementary Fig. 4c, the Burgers vector of the Shockley partial is 1/6[11-2], and its edge segment is in [1-10] direction while its screw segment in [11-2] direction. According to the disclination theory[2], the intrinsic stress field of a five-fold twin in a cylindrical NW can be expressed in Cartesian coordinates (x,y,z) shown in Supplementary Fig. 4 as

$$
\begin{aligned}
\sigma_{xx} &= \frac{G\omega}{2\pi(1-\nu)}\left(\frac{1}{2}\ln\frac{x^2+y^2}{R^2}+\frac{y^2}{x^2+y^2}\right)\\
\sigma_{yy} &= \frac{G\omega}{2\pi(1-\nu)}\left(\frac{1}{2}\ln\frac{x^2+y^2}{R^2}+\frac{x^2}{x^2+y^2}\right)\\
\sigma_{xy} &= -\frac{G\omega}{2\pi(1-\nu)}\frac{xy}{x^2+y^2}\\
\sigma_{zz} &= \frac{G\omega\nu}{2\pi(1-\nu)}\left(\ln\frac{x^2+y^2}{R^2}+1\right)
\end{aligned}
\quad (1)
$$

where R is the radius of the NW, ω the disclination angle, G the shear modulus, and ν Poisson's ratio. The Peach-Koehler force **F** acting on a dislocation segment with Burges vector of **b** and line vector of **ξ** by the stress field **σ** of a wedge disclination is expressed as

$$\mathbf{F} = \boldsymbol{\xi}\times(\boldsymbol{\sigma}\bullet\mathbf{b}) \quad (2)$$

the project of which is

$$F_b = [\boldsymbol{\xi}\times(\boldsymbol{\sigma}\bullet\mathbf{b})]\bullet\mathbf{b} \quad (3)$$

in the direction of the Burgers vector ***b*** and

$$F_y = [\boldsymbol{\xi}\times(\boldsymbol{\sigma}\bullet\mathbf{b})]\bullet\vec{e}_y \quad (4)$$

in the y direction. Substituting Eq. S1 and components of ***b*** and ***ξ*** into Eqs. S3 and S4, we obtain,

$$F_b = \frac{\sqrt{2}}{18}\xi_y(\sigma_{xx}-\sigma_{zz}) - \frac{1}{18}(2\xi_z+\sqrt{2}\xi_x)\sigma_{xy} \quad (5)$$

and

$$F_y = -\frac{1}{3}\xi_z\sigma_{xx} - \frac{\sqrt{2}}{6}\xi_x\sigma_{zz} \quad (6)$$

Note that ν=0.37 for silver and $\left|\frac{y}{x}\right| < \tan(\frac{70.53^\circ}{2})$ in the grain shown in Supplementary Fig. 4a. We obtain,

$$
\begin{aligned}
\sigma_{xx}-\sigma_{zz} &= \frac{G\omega}{2\pi(1-\nu)}\left(\frac{1}{2}\ln\frac{x^2+y^2}{R^2}+\frac{y^2}{x^2+y^2}-\nu\ln\frac{x^2+y^2}{R^2}-\nu\right)\\
&= \frac{G\omega}{2\pi(1-\nu)}\left(0.13\ln\frac{x^2+y^2}{R^2}-0.04\right) < 0
\end{aligned}
\quad (7)
$$

If the dislocation segment has an edge character, i.e. ξ is normal to $\boldsymbol{b}$, $\boldsymbol{\xi}$ =1/2[1-10], then

$$F_b = \frac{\sqrt{2}}{18}(\sigma_{xx} - \sigma_{zz}) < 0 \tag{8}$$

$$F_y = 0 \tag{9}$$

If the dislocation segment has a screw character, i.e. $\boldsymbol{\xi}$ is parallel to $\boldsymbol{b}$, or $\boldsymbol{\xi} = \boldsymbol{b}$, then

$$F_b = 0 \tag{10}$$

$$F_y = \frac{\sqrt{2}}{18}(\sigma_{zz} - \sigma_{xx}) > 0 \tag{11}$$

In both cases, the Peach-Koehler force exerted on the dislocation by the five-fold twin is repulsive, indicating that the intrinsic stress field favors dislocation retraction.

Supplementary Note 2 | Influence of vacancy on dislocation nucleation

In our simulations, the stress relaxation is highly sensitive to nanoscale imperfections (vacancies and dislocations) in the simulated samples. The simulations indicate that the stress relaxation is induced by dislocation nucleation facilitated by aggregation and rearrangements of vacancies. To understand how vacancies assist partial dislocation nucleation, we calculated the generalized planar fault (GPF) curves of Ag at different vacancy concentrations, as shown in Supplementary Fig. 7a. The GPF curve represents the energy cost of rigidly shifting two semi-infinite blocks of crystal on a {111) plane along a <112> direction in this plane[3], as illustrated in the inset in Supplementary Fig. 7a. The first peak of the GPF curve corresponds to the unstable stacking fault energy γ_{usf}, the energy barrier associated with the nucleation of leading partials. The valley of GPF curve denotes the stable stacking fault energy γ_{sf}, which determines the width (i.e. inter-spacing between leading and trailing partials) of an extended full dislocation in FCC metals. Previous studies[3,4] showed that at the nanoscale the nucleation of trailing partial dislocation is closely related to the relative difference between γ_{usf} and γ_{sf}, i.e. γ_{sf}/γ_{usf}. Smaller γ_{sf}/γ_{usf} (<1) indicates less prevalence of full dislocations as the trailing partial requires a larger nucleation barrier. In this case, partial dislocation slip becomes energetically favorable and dominates the plastic formation. For Ag, the ratio of γ_{sf}/γ_{usf} is only 0.16, much smaller than 1, therefore the slip of leading partials is a governing mechanism during plastic deformation. Supplementary Figs 7b-d shows the variations of γ_{usf}, γ_{sf} and γ_{sf}/γ_{usf} with vacancy concentration, respectively. It is seen in

Supplementary Figs. 7b-c that both γ_{sf} and γ_{usf} decrease as the vacancy concentration rises, implying that the vacancy can reduce the nucleation barrier for leading partials. The reduction of γ_{sf}/γ_{usf} in Supplementary Fig. 7d suggests that the slip of partial dislocations becomes a more dominating plastic deformation mechanism at higher vacancy concentration. This explains our observation that the stacking faults remain in the grain interiors even after the partials are absorbed by the twin boundaries.

In addition, we performed a simple simulation to examine the influence of vacancy on dislocation nucleation. The simulated sample is a 20×20×20 nm^3 Ag block with free surfaces in the [001] direction, as shown in Supplementary Fig. 8a. Periodic boundary conditions are imposed on [110] and $\left[\bar{1}10\right]$ directions. The sample is initially relaxed and equilibrated at 800 K using the Nosé-Hoover thermostat and barostat, and then stretched in the *y* direction at a constant strain rate of 10^8/s under NVT ensemble. To find the minimum strain corresponding to the first dislocation nucleation, we load the sample to different strains, relax the structure for 1 ns while the strain is held fixed, and then check whether there is dislocation nucleation. We run such kind of simulations for a perfect sample and a defective one with 1% vacancy. The results indicate that the first partial dislocation nucleates from the free surface at 4.15% strain in the vacancy-free sample, while such event occurs at 2.50% strain in the sample with vacancy concentration of 1%. This result indicates that the presence and aggregation of vacancies promote partial dislocation nucleation, as the nucleation barrier for leading partials is lowered by vacancy.

Supplementary Note 3 | Comparison of bi-crystalline samples with a twin boundary and those with tilting and mixed-type grain boundaries

In additional to three cases in Fig. 4, we also simulated bi-crystalline NWs with different types of grain boundaries (GBs) to further explore whether the observed fully recoverable behavior is closely connected to TBs. We selected a tilt GB with mis-orientation of 7°, a tilt GB with mis-orientation of 45° and a tilt-twist mixed GB to repeat the atomistic simulations of relaxation. In the case of the low-angle tilt GB, partial dislocation nucleated from surface can easily transverse through the GB during relaxation, as shown in Supplementary Fig. 9a. Such low-angle tilt GB cannot impede dislocation slip, because it consists of a sparse array of edge dislocations. During unloading, dislocation cannot move backwards because it has escaped out of the sample. As a

result, the stacking fault remains in the sample and no strain recovery is observed in this case. For the high-angle tilt GB, dislocations are sufficiently blocked by the GB and firmly trapped in it (Supplementary Fig. 9b). In this case, dislocations are locked to the GB and cannot retract back upon unloading, leading to irreversible plastic deformation. The same phenomenon was observed in the case of the tilt-twist mixed GB (Supplementary Fig. 9c). Comparing these results with those in Fig. 4, we find that only the TB can efficiently block dislocation motion and simultaneously ensures dislocation retraction upon unloading. This indicates that TB plays a unique and critical role in the observed fully strain recovery phenomenon.

Supplementary Note 4 | Characteristic time for relaxation

Stress relaxation in the penta-twinned nanowire can be characterized by the following equation,

$$\sigma(t) = \sigma_0 + \sigma_1 \exp\left(-t/\tau_\varepsilon\right) \tag{12}$$

where τ_ε is the relaxation time, σ_0 and σ_1 are constants. Based on this equation, we can fit all stress relaxation results from experiments and atomistic simulations. Supplementary Fig. 11 shows comparisons between simulation results and fitting curves. Supplementary Tables 1 and 2 summarize the relaxation time for all cases of experiments and simulations, respectively. For experimental data, the unrelaxed moduli for NW samples with diameters of *D*=112 nm and 104 nm are very close to the bulk modulus of Ag, while the unrelaxed moduli for *D*=48 nm sample is larger than the bulk value possibly due to the effects of surface stress and/or measurement error in the NW diameter and cross-sectional shape. Of course, there exist 10~11 orders of magnitude differences in the relaxation time between experiments and simulations due to different vacancy concentrations, sample dimensions and temperature. Interestingly, it is observed from Tables S1 and S2 that the characteristic relaxation time τ_ε increases as the sample diameter *D* is reduced. Such dependence of relaxation time on sample size can be explained by the damping effect of dislocation nucleation and motion.

The relaxation time characterizing this time-dependent behavior associated with dislocation activities can be expressed in the form of an Arrhenius equation[6],

$$\tau_\varepsilon = \tau_0 \exp\left(\frac{Q}{k_B T}\right) \tag{13}$$

where τ_0 is a constant, k_B the Boltzmann constant, T the temperature and Q the activation energy for dislocation nucleation and/or motion. In nucleation/motion of a dislocation loop, a critical condition is achieved when the activation energy is balanced by the work done on the dislocation by the applied stress σ. Therefore, the activation energy is proportional to the applied stress σ and can be expressed as,

$$Q = s\sigma bA \tag{14}$$

where b is the Burgers vector of a partial dislocation, A the area swept by the dislocation and s the Schmid factor. Combining Eqs. (S13) and (S14), we obtain

$$\tau_\varepsilon = \tau_0 \exp\left(\frac{s\sigma bA}{k_B T}\right) \tag{15}$$

For dislocation nucleation and motion in the penta-twinned NWs, the repulsive stress from twin boundaries must be overcome. Previous study on nanotwinned NWs[7] has shown that the applied stress for dislocation nucleation is inversely proportional to the sample diameter, i.e.

$$\sigma \propto 1/D \tag{16}$$

Combining Eqs. (S14) and (S15), we obtain an approximate relation between the relaxation time and sample diameter as follows,

$$\tau_\varepsilon \propto \exp\left(\frac{\alpha}{D}\right) \tag{17}$$

where α is just a factor. From the above equation, it is seen that the relaxation time depends on the sample diameter. As the sample diameter decreases, the relaxation time increases. This prediction is in good agreement with both experimental and simulation results.

Supplementary References

1 Wu, J. Y., Nagao, S., He, J. Y. & Zhang, Z. L. Role of five-fold twin boundary on the enhanced mechanical properties of fcc Fe nanowires. *Nano Lett.* **11**, 5264-5273, (2011).

2 Li, J. C. M. Disclination model of high angle grain-boundaries. *Surf. Sci.* **31**, 12-26 (1972).

3 Van Swygenhoven, H., Derlet, P. M. & Froseth, A. G. Stacking fault energies and slip in nanocrystalline metals. *Nature Mater.* **3**, 399-403, (2004).

4 Froseth, A., Derlet, P. & Van Swygenhoven, H. Dislocations emitted from nanocrystalline grain boundaries: nucleation and splitting distance. *Acta Mater.* **52**, 5863-5870 (2004).

5 Filleter, T. et al. Nucleation-controlled distributed plasticity in penta-twinned silver nanowires. *Small* **8**, 2986-2993, (2012).

6 Nowick, A. S. Anelastic relaxation in crystalline solids. *Elsevier*, 1972.

7 Deng, C. & Sansoz, F. Repulsive force of twin boundary on curved dislocations and its role on the yielding of twinned nanowires. *Scripta Mater.* **63**, 50-53 (2010).
